# Preparation of an Acridinium Ester-Labeled Antibody and Its Application in GoldMag Nanoparticle-Based, Ultrasensitive Chemiluminescence Immunoassay for the Detection of Human Epididymis Protein 4

**DOI:** 10.3390/mi8050149

**Published:** 2017-05-07

**Authors:** Ting Ma, Mengdan Zhang, Yinsheng Wan, Yali Cui, Le Ma

**Affiliations:** 1 College of Life Sciences, Northwest University, Xi’an 710069, China; mating1988527@sina.com; 2 National Engineering Research Center for Miniaturized Detection Systems, Xi’an 710069, China; mengdan0116@sina.com; 3 Department of Biology, Providence College, Providence, RI 02912, USA; YSWAN@providence.edu

**Keywords:** GoldMag particles, chemiluminescence immunoassay, acridinium ester, labeling, human epididymis protein 4

## Abstract

An ultrasensitive and rapid sandwich-type chemiluminescence immunoassay (CLIA) was developed for the clinical determination of human epididymis protein 4 (HE4) in human serum, using GoldMag nanoparticles as solid phase and acridinium ester (AE) as chemiluminescence system (GMP-CLIA). The process of AE labeling antibodies was systematically studied and evaluated. The effect of varies factors such as molar ratio of AE to antibodies, labeling time, and the components of elution buffer and trigger solution were optimized. Under the selected conditions, AE labeling experiments were successfully performed with the average labeling efficiency of 1.92 ± 0.08, and antibody utilization rate of 69.77 ± 1.19%. Antibody activity remained unchanged after labeling. The established GMP-CLIA method can detect HE4 in the range of 0.25–50 ng·mL^−1^ (10–2000 pM) with a detection limit of 0.084 ng·mL^−1^ (3.36 pM). The sensitivity has reached a high level, comparable with the current commercial detection kits. This proposed method has been successfully applied to the clinical determination of HE4 in 65 human sera. The results showed a good correlation with a clinical method, microplate-based chemiluminescence enzyme immunoassay (CLEIA), with the correlation coefficient of 0.9594.

## 1. Introduction

Immunoassay has been extensively applied in areas of clinical diagnostics [1,2]. Compared with the traditional technologies, such as radioimmunoassay (RIA) and enzyme-linked immunosorbent assay (ELISA), a novel technology which is based on chemiluminescent (CL) reaction, the chemiluminescent immunoassay (CLIA), has attracted much attention during the last decade. Due to the related wide dynamic range, high sensitivity, and low background, this technology has quickly become the main approach in clinical immunological analysis [3,4].

The sandwich-type is commonly-used mode for CLIA [5,6,7]. Lumimol and horseradish peroxidase (HRP) are most widely used as the CL label and catalyst, respectively [8,9]. In the majority of sandwich-type methods, a microplate is always used as an immobilization support [10]. Based on these, many of the classical biomarkers related to diseases in human blood have been detected accurately [11,12,13], which have greatly promoted the development of clinical diagnostic studies. However, challenges remain. The presence of enzymes usually increase the background of the reaction, and the separation and cleaning processes based on the microplate during immunoreaction are always complex and time-consuming [14]. With the development of personalized medicine, early clinical diagnosis becomes particularly important. Faster and more sensitive methods for detecting biomarkers is in great demand.

Magnetic particles, which are a new group of solid-phase carriers, have been utilized in clinical immunoassay [15,16]. With the micron- or nano-scale iron oxide as the core component, magnetic particles can rapidly aggregate under an external magnetic field [17]. When the external magnetic field is removed, the magnetic particles will be re-suspended in solution, which greatly reduces the cleaning time and is easy to automate. On the other hand, the bio-functionalized surface of particles can immobilize a large amount of biomolecules, which is much more than what a microplate can immobilize.

Our group first reported the preparation of Fe_3_O_4_/Au assembled particles in 2002 [18], named GoldMag particles, which have good superparamagnetism and biocompatibility. These unique particles have been successfully used in the field of magnetically-targeted drug delivery, nucleic acid purification and individual genotyping these years [19,20,21,22]. In our previous work, a GoldMag particle-based CLIA method for the detection of high-sensitive C-reactive protein (hsCRP) in human serum was developed, in which HRP-Luminol-H_2_O_2_ system was used [23]. However, the attachment of luminol to protein would lead to a decline in luminescence activity [24].

Acridinium ester (AE) is one of the novel chemiluminescent reagents developed ten years ago, which independently emits light in the presence of H_2_O_2_ and NaOH without the participation of an enzyme, and reaches its maximum CL only in 0.4 s [25]. More importantly, the labeled proteins have a minimal influence on AE mediated luminescence intensity [26]. Based on these advantages, commercialized AE labeling kits, such as acridinium protein labeling kits from Cayman and Enzo Life Sciences, has been commercialized quickly and applied in laboratory studies. Although these kits have been widely used in AE labeling on a variety of biomolecules, such as antibodies, peptides, and nucleic acids, the relatively low stability and efficiency for different types of antibodies limits its application in the development of our detection system. On the other hand, current evaluation methods for AE labeling include a spectrophotometry method for quantifying the concentration of AE on antibodies (the labeled product is treated with concentrated hydrochloric acid to form a colored acridine ester salt which absorbs light at 367 nm). Users have to perform relatively complicated calculations to eliminate the interference to antibody quantification (the protein has a maximum light absorption at 280 nm). A more convenient assessment method should be established.

**Figure 1:**
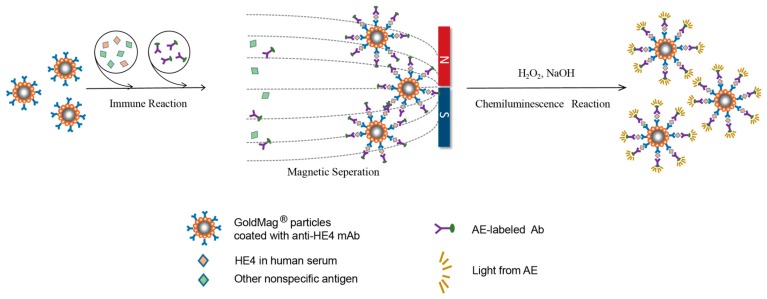
Schematic illustration of established sandwich-type GMP-CLIA method.

In this work, AE labeled-HE4 antibodies were prepared by using an improved labeling method with higher efficiency. By combining the AE-labeled antibodies with magnetic particles, we developed a detection system (GMP-CLIA) for the determination of human epididymis protein 4 (HE4), which is considered as a new specific biomarker of ovarian cancer [27,28]. The scheme of proposed GMP-CLIA method is illustrated in Figure 1. The primary antibody, mouse-anti-human HE4 was first immobilized on a magnetic nanoparticle, and then the antigen (human HE4) and the AE-labeled second antibody were conjugated successively to form a sandwich-type immunocomplex. CL was carried out in a solution containing NaOH and H_2_O_2_. The relative light unit (RLU) obtained from AE chemiluminescence was proportional to the concentration of the HE4 antigen. This detection system is faster and simpler than the traditional methods, such as ELISA and CLEIA, exhibits high sensitivity which is comparable to the Roche electrochemical luminescence assay (ECLIA), and shows great application potential in clinical detection.

## 2. Materials and Methods

### 2.1. Materials

Acridinium NHS ester was purchased from Cayman Chemical (Ann Arbor, MI, USA). Mouse anti-HE4 monoclonal antibodies 3C24 (capture) and 2B13 (detection) were obtained from HyTest Ltd. (TurKu, Finland), and HE4 recombinant antigen was obtained from Sino Biological Inc. (Beijing, China). Bovine serum albumin (BSA), DL-lysine hydrochloride and HRP-goat anti-mouse antibodies were from Sigma-Aldrich (St. Louis, MI, USA). The Sephadex G-25 desalting column was purchased from GE Healthcare Life Sciences (Pittsburgh, PA, USA). GoldMag particles were supplied by the National Engineering Research Center for Micro Detection System (Xi’an, China). The commercial HE4 CLEIA kit was purchased from Connery (Gothenburg, Switzerland). All aqueous solutions were prepared using water purified with an ultrapure water system (Millipore, Billerica, MA, USA).

### 2.2. Preparation of AE-Labeled Antibodies

Anti-HE4 antibody (2B13) solution (250 μg) was placed in 2 mL centrifuge tubes and mixed well with labeling buffer (0.1 M Phosphate buffered saline (PBS) containing 0.15 M NaCl, pH 8.0). Then a certain volume of 0.5 mM AE solution was added in the dark, and the final volume was 1000 μL. The antibody was labeled in the dark at 180 rpm in a constant-temperature incubator (25 °C). Next, 100 μL of 5% dl-lysine hydrochloride solution was added into the tube and incubated in the constant-temperature incubator at 180 rpm (25 °C) for 30 min. Then the reaction was terminated. The labeled mixture was loaded to the pre-balanced Sephadex G-25 desalting column (1.6 × 2.5 cm) for elution and collection of target antibodies. PBS (0.1 M) containing 0.15 M NaCl (pH 6.3) was used as elution buffer. The eluted components were collected with 500 μL in each tube and 24 tubes were obtained.

### 2.3. Evaluation of the Labeling Effect

For the 24 tubes of elutes, RLU were determined, respectively. Two microliters of each elute was placed into the special detection tube (12 × 60 mm), then the trigger solution was added automatically and the RLU from the emitting light of the AE was collected almost at the same time by a BHP9507 chemiluminescence analyzer (Hamamatsu Photon Techniques, Beijing, China). Antibody concentration in 24 tubes of elutes were also determined by using a NanoDrop 2000c ultramicrospectrophotometer (Thermo Fisher Scientific, Waltham, MA, USA). In order to observe each elute clearly, the number of tubes was plotted on the *X*-axis, and the RLU and the concentration of antibodies measured on the *Y*-axis, respectively. The overlap between the RLU peak and the concentration peak just corresponds to the AE-labeled antibodies. Finally, the elutes containing AE-labeled antibody were mixed well, and added with 0.1% BSA and 0.1% ProClin 300, stored at 4 °C for short time, or added with 50% glycerol and stored at −20 °C for long-term storage.

The labeling efficiency was defined as the ratio of molar concentration of AE to that of labeled antibody in the elute mixture above. “Standard curve method” was used to determine the molar concentration of AE in every elute sample, where the RLU value was the *Y*-axis and the gradient concentration of AE standard solution was the *X*-axis.

The utilization rate of antibodies was defined as the mass percentage of labeled antibody to total antibody in advance (250 μg). The amount of labeled antibodies was calculated by the concentration of the labeled antibodies and their elution volume.

Antibody activity before and after labeling was assessed by ELISA. Anti-HE4 antibodies before and after labeling was adjusted to the same concentration firstly. HE4 antigen was dissolved in 0.1 M PBS to achieve the final concentration of 5 μg/mL. The plate was coated with antigen solution at 100 μL/well at 2–8 °C overnight. The plate was washed with PBST the next day and sealed with sealing solution for 2 h at 37 °C. After washing with PBST, the anti-HE4 antibody before and after labeling was diluted from 10^3^ to 10^8^ times with elution buffer. The sample was added at 100 μL/well. The eluent buffer was used as the negative control. The cells were incubated at 37 °C for 1 h and added with 1:2000 HRP-goat anti-mouse antibody at 100 μL/well. After incubation at 37 °C for 1 h and washing with PBST, the TMB substrate buffers A and B were added at 50 μL/well for reaction away from light for 15 min. The stopping buffer was added at 50 μL/well to determine A450, which was measured by a WD-2102A automatic microplate reader (Beijing Liuyi, Beijing, China).

### 2.4. GMP-CLIA for HE4

In sandwich-type immunoassay, two antibodies are often utilized for target antigen detection. We used anti-HE4 antibody (3C24) as the capture antibody and the AE-labeled anti-HE4 antibody (2B13) as the detection antibody. These two distinct monoclonal antibodies can recognize different epitopes on HE4 molecules. First, we prepared GoldMag particles by following the procedures reported previously [18]. And then, immobilized anti-HE4 capture antibody onto GoldMag particles to form the anti-HE4-GMPs. GoldMag particles (1 mg) were incubated with 50 μg of anti-HE4 antibody (3C24) in 200 μL of Tris-HCl buffer (0.02 M, pH7.4) at 180 rpm, 37 °C for 30 min. After washing with PBST, 1 mL of sealing buffer (0.1 M PBS containing blocking agents) was added for reaction at 180 rpm, at 37 °C for 2 h. Then, particles coated with antibodies were washed with PBST and resuspended with 1 mL of storage buffer (0.1 M PBS containing BSA and ProClin 300) and the final concentration of particles was made to be 1 mg/mL for use. Next, the HE4 antigen was diluted to concentration gradient of 50 ng·mL^−1^, 40 ng·mL^−1^, 30 ng·mL^−1^, 20 ng·mL^−1^, 10 ng·mL^−1^, 5 ng·mL^−1^, 2.5 ng·mL^−1^, 1 ng·mL^−1^, 0.5 ng·mL^−1^, and 0.25 ng·mL^−1^. AE-labeled anti-HE4 antibodies prepared in advance were diluted by 100 times. Finally, 30 μL of anti-HE4 Ab-GMPs, 50 μL of HE4 antigen of each concentration, and 100 μL of diluted AE-anti-HE4 were added into the special detection tube one time (the final concentration of particles was 16.6 mg/mL). Then, the resulting mixture was incubated in a constant-temperature incubator (Zhicheng, China) with gentle shaking, at 180 rpm and 37 °C for 40 min. When the reaction finished, the products were washed with PBST three times. The supernatant was discarded by magnetic separation. Finally, 100 μL of trigger solution was automatically loaded into each tube. The RLU of each tube was detected immediately by using BHP9507 chemiluminescence analyzer (Hamamatsu Photon Techniques, Beijing). The standard curve of the antigen concentration vs. RLU was plotted, with the concentration of the HE4 antigen on the *X*-axis and the RLU on the *Y*-axis.

### 2.5. Detection Limit and Functional Sensitivity Measurement

The RLU of the negative sample was measured 20 times. The mean (M) and the standard deviation (SD) were calculated to obtain the value of M + 2SD. The M + 2SD value was treated as the RLU to be substituted into the standard curve equation. Thus, the corresponding concentration, i.e., the detection limit, was calculated. The HE4 reference substance with the low concentration of 0.20 ng·mL^−1^, 0.25 ng·mL^−1^, and 0.30 ng·mL^−1^ was measured, respectively, 20 times. The variation coefficient (CV) of the measurements was calculated. The lowest concentration with a CV smaller than 20% represented the functional sensitivity of the HE4 detection system.

### 2.6. Clinical Application and Analysis

Clinical samples used in this study were obtained from the First Affiliated Hospital of Zhejiang University Medical College. HE4 of 65 clinical serum samples were determined by using the proposed method, and the results were compared with that obtained by the clinical method (microplate-based CLEIA). Linear correlation analysis was performed. The regression equation and correlation coefficients were used to judge the correlation between two methods. In order to determine the consistency, the Bland-Altman method was used for stochastic effect analysis on their differences, with the average of concentration determined by two methods as the *X*-axis, and relative error (the ratio of the difference to average) as the *Y*-axis. Finally, the mean relative error and the 95% confidence interval were determined.

## 3. Results

### 3.1. Characteristics of AE-Labeled Antibodies

**Figure 2:**
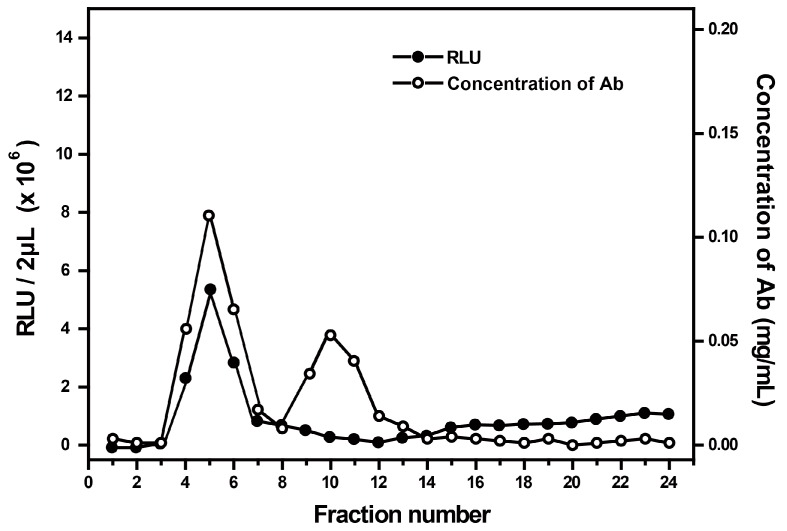
Relative light units (RLU) and concentration of antibody (mg·mL^−1^) for each elute tube. Two-hundred fifty micrograms of antibodies were incubated with a five-fold molar amount of AE at 25 °C for 20 min in the dark. Labeling products were applied to a Sephadex G-25 desalting column and 24 tubes of elutes were collected.

First, we incubated the antibodies with AE for labeling by following preparing procedure described in Section 2.2, and then loaded the resulting mixture onto the Sephadex G-25 for elution and collection of target antibodies. To characterize AE-labeled antibodies, RLU and protein concentration from 24 tubes of elutes were measured respectively. As shown in Figure 2, the RLU peak of AE obviously appeared at the position of tubes 4, 5 and 6. Since the antibodies labeled with AE had the largest molecular weight, they should be first eluted. Thus, the RLU peak at the position of tubes 4, 5 and 6 was contributed by the AE that was labeled onto antibodies. The peak value indicated that a large amount of labeled antibodies were eluted as the major component collected. After that, RLU declined until tube 12, and then appeared slow rise. The decline of RLU suggests that most of the labeled antibody are eluted. A slow rise of RLU (tubes 13–24) indicated that the free AE molecules closely bound to the Sephadex G-25 column were gradually eluted. There are two peaks on the concentration curve. The first peak appeared at the position of tubes 4, 5 and 6, which overlap with the only one RLU peak, while the second appeared alone at the position of tubes 8, 9, 10, 11 and 12. Since there was no interference caused by other impure proteins, it can be concluded with certainty that the first peak was contributed by AE-labeled protein. Correspondingly, the second peak represents unlabeled antibody. Thus, products in tubes 4, 5, and 6 were mixed together for future use.

### 3.2. Optimization of the Labeling Conditions

In order to obtain the stable and highly-efficient AE-labeled antibodies, we optimized some critical conditions, including the molar ratio of AE to antibody, labeling time, and composition of trigger solution, which might affect the AE labeling process.

**Figure 3:**
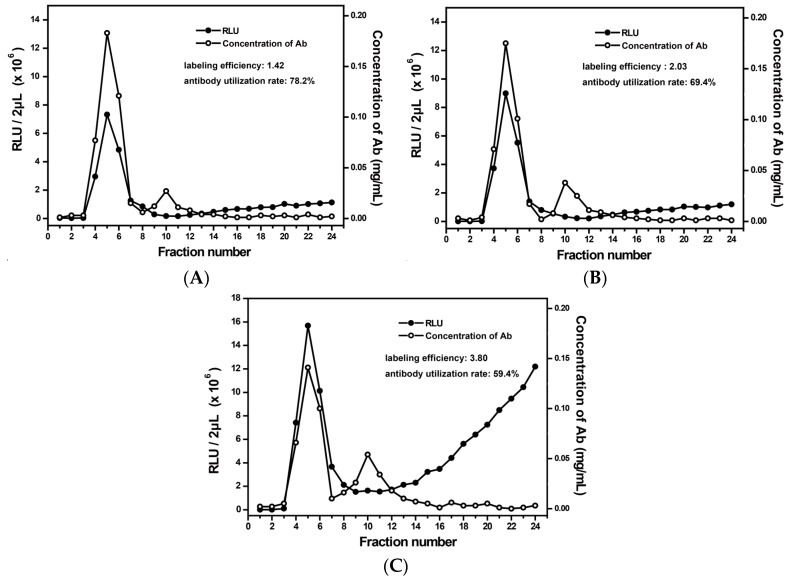
The effect of the molar ratio of AE to antibodies on RLU and the concentration of each elute. (**A**–**C**) correspond to a series of molar ratios respectively (5:1, 15:1, and 35:1).

A too-low dosage of AE might cause an incomplete reaction with the antibodies and, hence, a low labeling efficiency. In contrast, an excess dosage might cause precipitation, which would affect the labeling efficiency [29]. Thus, three dosages of AE (high, medium, and low) were selected, corresponding to the molar ratio (AE:antibody) of 5:1, 15:1, and 35:1, respectively. As shown in Figure 3, when the molar ratio was 35:1 (Figure 3C), the excess dosage of AE resulted in an obvious RLU-increasing trend after the position of tube 12. This is due to a large amount of free AE that was eluted and not used during the labeling process. The corresponding antibody utilization rate is only 59.4%. When the molar ratio was 5:1, although the antibody utilization rate was 78.2%, the labeling efficiency was relatively low (Figure 3A). Finally, 15:1 was chosen as the optimal condition (Figure 3B). Under this condition, the AE labeling efficiency was about 2.03 and the antibody utilization rate was 69.4%.

**Figure 4:**
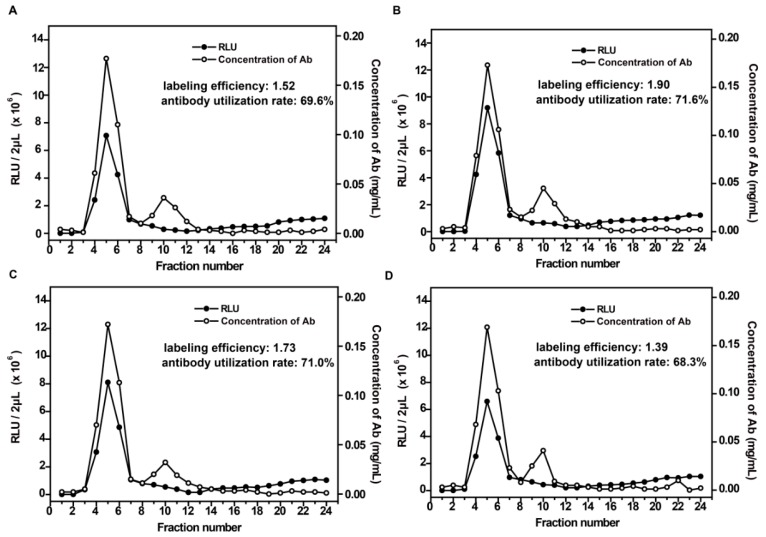
The effect of labeling time on the RLU and concentration of components in each elute tube. (**A**–**D**) correspond to a series of durations required for the labeling reaction (10, 20, 30, and 60 min, respectively).

To determine the optimal labeling time, the labeling reaction time of 10 min, 20 min, 30 min, and 1 h were chosen, respectively. As shown in Figure 4, labeling efficiency achieved a maximum when the labeling time was 20 min (Figure 4B), while the antibody utilization rate did not change significantly under different labeling times. The results indicated that either a too short or a too long reaction time would affect the labeling efficiency.

**Figure 5:**
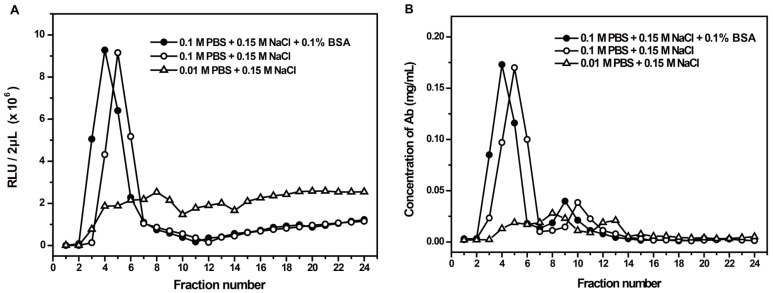
Comparison of different elution buffers. (**A**) and (**B**) respectively correspond to the RLU and concentration of Ab in each elute tube under different elution buffer conditions.

Generally, PBS containing BSA or human IgG was used as the elution buffer for reducing the physical adsorption loss of target protein. However, the presence of BSA or IgG might interfere the determination of target protein concentration by using spectrophotometry method in this study. Therefore, we measured the possible differences between protein-free PBS buffer and PBS buffer containing 0.1% BSA when they were used as elution buffer, respectively. PBS buffer with low ionic concentration was used as control. As shown in Figure 5, the absence of BSA in elution buffer did not affect the elution amount of the labeled- and unlabeled-antibodies.

The CL behavior of AE can be initiated in the presence of H_2_O_2_ and NaOH. In an alkaline environment, the C9 position of the acridine ring binds to the hydroxide ions in the solution, forming the pseudobase, which is unfavorable for the chemiluminescence of acridinium ester [30]. There have been reports suggesting that the addition of HNO_3_ before the CL reaction could create an acidic environment, which inhibits the formation of pseudobase and, furthermore, a suitable surfactant could increase the CL intensity [31]. Two microliters, at high concentration (6.25 × 10^−7^ mol·L^−1^) and low concentration (1.56 × 10^−7^ mol·L^−1^), of AE solution was excited respectively by different trigger solutions, and RLUs were collected. Finally, a suitable trigger solution was selected by comparison of the RLU value. As shown in Table 1, when 0.1 M HNO_3_ was added before NaOH, RLU from the AE solution was 5–10 times more than that without HNO_3_ addition. Moreover, when 2% Tween-20 or Triton-100 was added into solution B, RLU value increased in various degrees. Thus, solution A, containing 0.1% H_2_O_2_ and 0.1 M HNO_3_, and solution B, containing 0.25 M NaOH and 2% Triton-100, were selected as the trigger solution.

Finally, the optimal conditions for AE labeling and CL reaction were optimized as follows: 15:1 molar ratio of AE to antibodies, 20 min labeling time, 0.1 M PBS buffer (BSA-free) for elution, and a trigger solution added 0.1 M HNO_3_ and 2% Triton-100. It was noted that the activity of antibodies was also measured under different labeling efficiencies (data not shown), and no significant difference was found. The activity of antibodies after AE labeling were sufficient in the following detection.

### 3.3. Evaluation of the Labeling System

Based on the optimized conditions above, AE labeling experiments were performed three times. We further assessed the labeling system by using three parameters: labeling efficiency, utilization rate of antibodies, and antibody activity.

**Figure 6:**
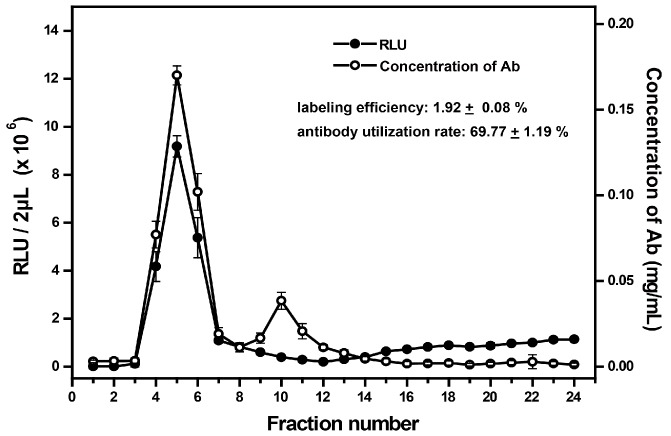
The average RLU and concentration of antibody for each elute. The average labeling efficiency and antibody use rate were 1.92 ± 0.08% and 69.77 ± 1.19%, respectively. Each point represents the mean value of triplicate determinations.

The labeling efficiency was defined as the ratio of molar concentration of AE to that of labeled antibodies in the elute mixture (4th, 5th, and 6th tubes). The antibody concentration and AE luminescence of all elutes from three experiments are shown in Figure 6. We measured RLU values for a series of gradient AE solutions, and used the linear correlation between them to further determine the AE concentration of the sample with known RLU. After calculation, the molar concentration of AE labeled on antibodies was 14.84 ± 0.21 × 10^−7^ M, and the molar concentration of labeled antibodies was 7.73 ± 0.13 × 10^−7^ M; thus, the labeling efficiency was 1.92 ± 0.08.

The utilization rate of antibody was defined as the percentage of AE-labeled antibody amount accounted for that of total antibody we provided in advance. The average concentration of labeled antibodies from the three-tube mixed elutes was determined to be 0.116 ± 0.002 mg·mL^−1^. The corresponding volume was 1500 μL and the total amount of antibody we provided was 250 μg, thus, the average utilization rate of antibody was 69.77 ± 1.19%.

For a comparison of antibody activity before and after labeling, an antibody titer was determined by using ELISA. Generally, an antibody dilution factor corresponding to an absorbance value of 2.1 times that of the background is used as the antibody titer. Thus, our results showed that the antibody titers before and after labeling were both about 1:10^7^ (Table 2). This means that the immunological activity of the labeled antibody was not influenced significantly by the labeling process.

Homogeneity of labeling process was determined finally. According to the separation principles of a Sephadex chromatographic column, the components would encounter different resistance when passing through the column because of their different molecular weight. Based on this, AE-labeled antibodies with larger molecular weight were first eluted and mainly in the 4th, 5th, and 6th tube, while free AE with minimal molecular weight was, finally, eluted. We wondered whether the antibodies in the front tubes would be labeled with more AE molecules. In fact, it was found that there was no significant difference in labeling efficiency of antibodies between the three tubes, which indicated that the labeling process was stable and homogeneous.

### 3.4. Establishment of GMP-CLIA for Detection of HE4

After successful preparation of AE-labeled antibodies, we next aimed to develop a CLIA system for detection of HE4, a new biomarker of human ovarian cancer. In the clinic, the significant HE4 concentration in human serum for diagnosis is about 3.5 ng·mL^−1^. In order to obtain a sufficiently high sensitivity that might be suitable for clinical diagnosis at the nanometer level, not only a strong signal source, but also an excellent immobilized carrier is needed. For the latter, GoldMag particles with a core-shell structure and large surface area-to-volume ratio were selected. Our previous work reported their application in quantitative detection of hsCRP in human serum, although the detection sensitivity was only achieved at the microgram level [23].

**Figure 7:**
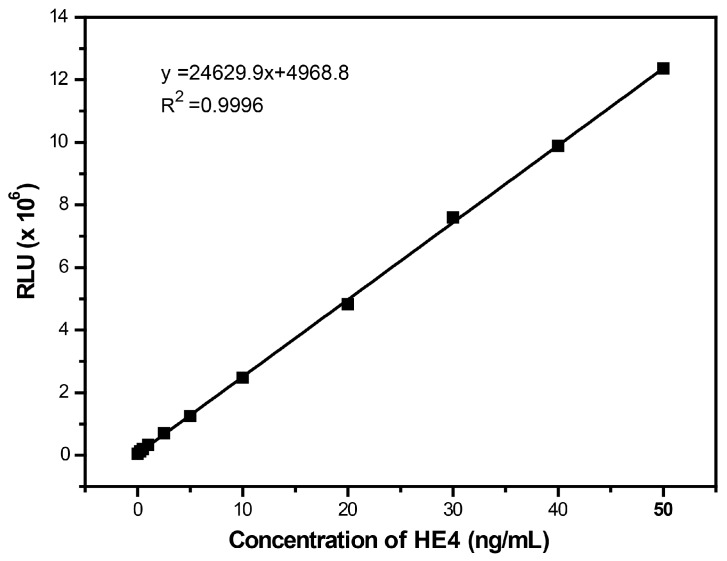
Calibration curve of CL intensity versus the concentration of HE4. Thirty microliters of magnetic particles coated with anti-HE4 antibody, 50 μL HE4 antigen solution, and 50 μL AE-labeled antibody solution were involved in the detection system. Immune reaction time, 20 min; trigger solution, A, 0.1% H_2_O_2_ + 0.1 M HNO_3_, B, 0.25 M NaOH + 2% Triton-100.

To establish the detection system, the amount of magnetic particle, antigen, antibody, and immune reaction time were optimized successively according to our previous report. The standard calibration curve was successfully obtained for RLU values against HE4 concentrations of 0.25–50 ng·mL^−1^. As shown in Figure 7, there is a good linearity and the square of the correlation coefficient is 0.9996. The detection limit and functional sensitivity of the present approach is 0.084 ng·mL^−1^ and 0.25 ng·mL^−1^, respectively (Table 3). The results demonstrated that the proposed method could be used for the determination of HE4. Moreover, the whole detection is finished in less than one hour, and the sensitivity of the present method is similar with that of commercially-available test kit from Roche (based on ECLIA) and higher than that from CanAg (based on ELISA).

### 3.5. Application in Human Serum Samples

**Figure 8:**
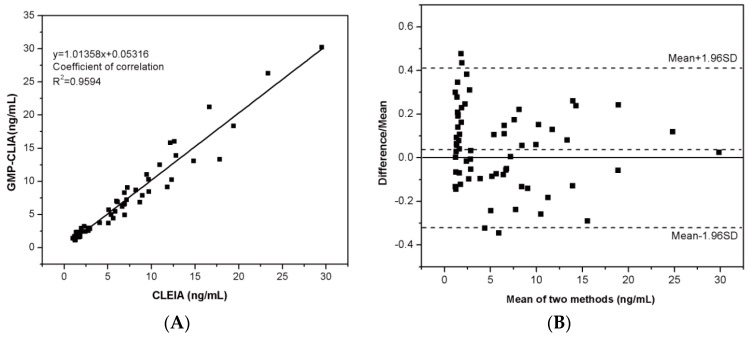
Comparison between results obtained using our method and the clinical method (microplate-based CLEIA). Sixty-five clinical human serums were detected respectively. (**A**) Correlation analysis; (**B**) Bland-Altman analysis. Horizontal dashed lines on either side of zero represent the 95% CIs (−0.31572–0.40818 ng·mL^−1^) for the difference between two methods.

To further assess the feasibility of established method for clinical application, 65 serum samples were analyzed, and the results of each co-responding serum sample obtained from the First Affiliated Hospital of Zhejiang University Medical College were used for comparison. As shown in Figure 8, there was good agreement between our method and clinical method. The linear regression equation was *y* = 1.0136*x* + 0.0532 and the square of the correlation coefficient was 0.9594. Bland-Altman analysis was further performed to judge their consistency. There existed an average deviation of 0.0462 ng/mL and most of the difference points were within the 95% confidence interval −0.31572 to 0.40818 ng/mL), suggesting a good consistency. Therefore, our developed GMP-CLIA method is applicable for the determination of HE4 in real samples, human serum.

## 4. Discussion

In this study, a CLIA method based on AE labeling and magnetic particles for quantitative detection of HE4 in human serum was developed. Aiming at the complicated evaluation method and instability of AE labeling in previous works, we optimized the conditions that may affect the labeling efficiency and CL behavior, including the molar ratio of AE to antibodies, labeling time, components of the elution buffer, and trigger solution. Here, we developed a simple and systematic evaluation method for AE labeling and, finally, confirmed that the labeled antibodies had superior performances for biological detection. On this basis, the sandwich-type CLIA detection system was established by using our unique GoldMag particles as the solid-phase carrier. The results showed that HE4 could be detected in the range of 0.25–50 ng·mL^−1^ (10–2000 pM) with a detection limit of 0.084 ng·mL^−1^ (3.36 pM). The established method was finally applied to quantitatively determine HE4 concentration in 65 human serum samples and compared with the results obtained from clinical method (microplate-based CLEIA). Correlation and consistency analysis suggest that GMP-CLIA method is accurate enough and applicable for the biodetection in real clinical serum samples. Our method shows a much higher sensitivity and reduced detection time in HE4 assay than traditional CLEIA and ELISA. Although there is no significant difference in sensitivity compared with ECLIA (Roche) (the highest sensitivity in the chemiluminescence detection field), a relatively simple device was crafted with our detection system, which makes it a high-accuracy, fast, and low-cost HE4 assay method for clinical application.

Due to the strong signal intensity of AE and amplification effect of magnetic particles, the established method has several obvious advantages. (1) This method greatly reduces the detection time. The entire test is finished within 1 h, whereas 2–3 h is required for the microplate CLEIA method; (2) Our method has high sensitivity and a wider detection range. In a magnetic field, proteins at low concentrations of HE4 can be enriched and the sensitivity can be enhanced. GMPs have a large surface area-to-volume ratio, and their application for antibody or antigen immobilization affords a linear detection range of 0.25–50 ng·mL^−1^ (10–2000 pM) which is sufficient for the detection of HE4 physiological (<140 pM, 3.5 ng·mL^−1^) and clinical levels (140–800 pM).

This work has demonstrated that GoldMag particles as the immobilized supporter could effectively amplify the CL of sandwich-type immunocomplex labeled with AE, which is quite suitable for developing a rapid, highly-sensitive commercial kit for the detection of biomarkers including, but not limited to, HE4 in clinical diagnosis.

## 5. Conclusions

A high-sensitivity, relatively simple and rapid CLIA has been developed for the clinical determination of HE4 in human serum, using AE chemiluminescence system combined with GoldMag magnetic particles. This assay provides apparent advantages and shows great potential in the clinical diagnosis.

## Figures and Tables

**Table 1:** Effect of different trigger solutions used in CL reaction of AE.

No	Trigger Solution	RLU_1_^a^	RLU_2_^b^
1	(A) 0.1% H_2_O_2_	17,239 ± 902	2266 ± 308
	(B) 0.25 M NaOH		
2	(A) 0.1% H_2_O_2_ + 0.1 M HNO_3_	180,503 ± 6011	13,266 ± 927
	(B) 0.25 M NaOH		
3	(A) 0.1% H_2_O_2_ + 0.1 M HNO_3_	484,743 ± 4813	55,142 ± 882
	(B) 0.25 M NaOH + 2% Tween-20		
4	(A) 0.1% H_2_O_2_ + 0.1 M HNO_3_	1,145,820 ± 18921	95,443 ± 730
	(B) 0.25 M NaOH + 2% Triton-100		

^a^ 6.25 × 10^−7^ mol/L AE solution in 0.1 M PBS, pH 6.3; ^b^ 1.56 × 10^−7^ mol/L AE solution in 0.1 M PBS, pH 6.3.

**Table 2:** Comparison of HE4 antibody activity before and after labeling.

Item	AE-Labeled Antibody						Non-Labeled Antibody						Blank
Dilution factor	10^3^	10^4^	10^5^	10^6^	10^7^	10^8^	10^3^	10^4^	10^5^	10^6^	10^7^	10^8^	/
A450	1.692	1.425	0.764	0.394	0.201	0.101	1.797	1.569	0.887	0.476	0.245	0.138	0.078

**Table 3:** Detection limit (DL) and functional sensitivity of the GMP-CLIA for HE4.

Detection Limit		Functional Sensitivity		
Mean (*n* = 20)	4231.70	Conc. of HE4 (ng/mL)	Mean (*n* = 20)	CV (%)
SD	784.48	0.20	9853	22
Mean + 2SD	5800.46	0.25	11,872	18
DL (ng/mL)	0.084	0.30	13,294	9
